# Inactivation of zearalenone (ZEN) and deoxynivalenol (DON) in complete feed for weaned piglets: Efficacy of ZEN hydrolase ZenA and of sodium metabisulfite (SBS) as feed additives

**DOI:** 10.1007/s12550-023-00486-2

**Published:** 2023-05-30

**Authors:** Sven Dänicke, Linn Carlson, Ann-Katrin Heymann, Angelika Grümpel-Schlüter, Barbara Doupovec, Dian Schatzmayr, Barbara Streit, Susanne Kersten, Jeannette Kluess

**Affiliations:** 1 Institute of Animal Nutrition, Friedrich-Loeffler-Institut (FLI), Federal Research Institute for Animal Health, 38116 Braunschweig, Germany; 2 DSM - BIOMIN Research Center, Tulln, Austria

**Keywords:** Pig, Deoxynivalenol, Zearalenone, Sodium metabisulfite, Zearalenone hydrolase

## Abstract

Female pigs respond sensitive both to DON and ZEN with anorexia and endocrine disruption, respectively, when critical diet concentrations are exceeded. Therefore, the frequent co-contamination of feed by DON and ZEN requires their parallel inactivation. The additive ZenA hydrolyzes ZEN while SBS inactivates DON through sulfonation. Both supplements were simultaneously added (**+**, 2.5 g SBS and 100 U ZenA/kg) to a control diet (CON−, 0.04 mg DON and < 0.004 mg ZEN/kg; CON+, 0.03 mg DON and < 0.004 mg ZEN/kg) and a *Fusarium* toxin contaminated diet (FUS−, 2.57 mg DON and 0.24 mg ZEN/kg; FUS**+**, 2.04 mg DON and 0.24 mg ZEN/kg). The 4 diets were fed to 20 female weaned piglets each (6 kg initial body weight) for 35 days; the piglets were sacrificed thereafter for collecting samples. Supplements improved performance and modified metabolism and hematology independent of dietary DON contamination. The mechanisms behind these changes could not be clarified and require further consideration. SBS reduced DON concentration in feed by approximately 20% and to the same extent in blood plasma and urine suggesting that no further DON sulfonate formation occurred in the digestive tract before absorbing DON in the upper digestive tract or that additionally formed DON sulfonates escaped absorption. DON sulfonates were detected in feces suggesting that unabsorbed DON sulfonates reached feces and/or that unabsorbed DON was sulfonated in the hindgut. The observed reduction rate of 20% was evaluated to be insufficient for feeding practice. Galenic form of SBS added to dry feed needs to be improved to support the DON sulfonation in the proximal digestive tract.ZenA was active in the digestive tract as demonstrated by the presence of its hydrolyzed none-estrogenic reaction products hydrolyzed ZEN (HZEN) and decarboxylated and hydrolyzed ZEN (DHZEN) both in feces, systemic circulation, and urine of group FUS+ compared to group FUS−. The presence of these hydrolysis products was paralleled by a significant decrease in high-estrogenic ZEN concentrations which, in turn, was related to a decrease in relative weights of uteri and ovaries when compared to group FUS−. Thus, ZenA was proven to be effective; both in terms of biomarkers and biological effects.

## Introduction

The *Fusarium* mycotoxins zearalenone (ZEN) and deoxynivalenol (DON) are common co-contaminants of feedstuffs and of complete feed for pigs (Schatzmayr and Streit 2013; Tolosa et al. 2021). These animals respond very sensitive to the presence of both toxins; mainly with a decreased feed intake and associated adverse down-stream effects, including live weight gain and health traits in the case of DON. ZEN, on the other hand, exerts estrogenic properties giving rise to clinical signs of hyperestrogenism and endocrine disruption which is particularly relevant for female pre-pubertal pigs (Döll and Dänicke 2011). To prevent those adverse effects on pigs, EU recommended that complete feed for pigs must not exceed 0.9 mg DON/kg and 0.1 mg ZEN/kg, respectively, at a reference dry matter (DM) content of 88% (European Commission 2006). DON and ZEN surveys have demonstrated that these feed safety levels are exceeded occasionally (Döll and Dänicke 2011). Besides preventive measures that minimize the formation of DON and ZEN in crops prior to harvest, inactivation strategies for contaminated feed are necessary. Based on the sensitivity of female young pigs, both to DON and ZEN and due to the co-occurrence of these toxins in contaminated feedstuffs, the challenge lies in the parallel inactivation of DON and ZEN. From a practical viewpoint, those strategies would be preferable which make use of feed additives supposed to inactivate the toxins while passing together with the ingesta through the digestive tract. Feed enzyme preparations capable of modifying the structure of the complementary toxin substrate in such a way that toxicity is markedly reduced are most promising based on their highly specific mode of action. The bacterial enzyme zearalenone hydrolase ZenA applied as a feed additive degrades ZEN in the gastrointestinal tract (Gruber-Dorninger et al. 2021, 2023). The enzymatic hydrolysis of ZEN with this enzyme was shown to release hydrolyzed zearalenone (HZEN), which partially converts to decarboxylated hydrolyzed zearalenone (DHZEN). Both ZEN degradation compounds were characterized by a significantly reduced estrogenic activity in vitro and in female pigs (Fruhauf et al. 2019). While ZEN exposure of the pigs resulted in a significantly enlarged reproductive tract the equimolar exposure of pigs to either HZEN or DHZEN failed to induce estrogenic activity. However, an investigation of the efficacy of ZenA in vivo requires feeding ZEN-contaminated diets in the absence and presence of the enzyme preparation and to monitor both toxicological endpoints, e.g., uterus weight and toxin residue levels including ZEN, HZEN, and DHZEN in physiological specimen indicative for systemic absorption and consequently reflecting their potential concentrations in target tissues.

In contrast to ZEN, a purified enzyme preparation for DON inactivation is currently not available although a microbial feed additive expressing DON hydrolyzing activity was authorized (European Commission 2013). Thus, feed additives which inactivate DON via other mechanisms need to be considered to cope with the DON co-contamination. Controversial results were reported for the efficacy of adsorbents for sequestration of DON both in vitro (Avantaggiato et al. 2005; Döll et al. 2004) and in vivo (Döll and Dänicke 2004). Sulfiting compounds such as sodium metabisulfite (SBS) and sodium sulfite (SoS) were shown to be effective in the inactivation of DON through the formation of different sulfonated compounds characterized by a low cytotoxicity and termed as DON-sulfonate 1, 2, and 3 (DONS1, DONS2, and DONS3) (Paulick et al. 2018; Schwartz et al. 2013). While this reaction is reproducible under hydrothermal (Dänicke et al. 2005; Rempe et al. 2013; Young et al. 1986) and wet preservation conditions (Paulick et al. 2015a; Dänicke et al. 2009), the effect was inconsistent when the substances were added to dry feed (Paulick et al. 2015b; Schwartz-Zimmermann et al. 2014a). While the bioavailability of DON was not substantially decreased when SBS was added to the contaminated diets just prior to wet feeding to pigs (Paulick et al. 2015b), a time-dependent decrease of DON concentration in air-dry contaminated feed was reported which appeared to be dependent on storage time, feed matrix, and SBS dose (Schwartz-Zimmermann et al. 2014b). Moreover, pelleting of DON-contaminated feed after short-time conditioning decreased DON concentration in feed and urinary DON excretion of pigs suggesting a reduced systemic DON availability (Frobose et al. 2017) although the effect of pelleting appeared to be dependent on SBS dose (Frobose et al. 2015).

Based on these previous results, it was hypothesized that ZenA effectively inactivates ZEN in vivo which is reflected in a reduced estrogenic activity compared to non-supplemented ZEN-contaminated feed along with a decreased systemic ZEN bioavailability as reflected by lower ZEN levels in blood and urine. Furthermore, simultaneously added SBS was hypothesized to inactivate DON to DON sulfonates in feed and/or mediated by the physico-chemical conditions of ingesta when passing through the digestive tract which should counteract the anorectic effects of DON and lead to lowered levels of intact DON in systemic circulation and urine. Based on these hypotheses, the aim of this study was to test the in vivo efficacy of ZenA and SBS when added to a ZEN and DON-contaminated diet based on both toxic effects and toxin residue levels in physiological specimens in female weaning piglets.

## Material and methods

### Experimental design and diets

To investigate the effectiveness of ZenA and SBS when added to dry feed, 4 diets were tested to cover a complete 2 × 2 factorial design with *Fusarium* toxin contamination (CON, background contaminated control feed; FUS, *Fusarium* toxin contaminated feed) and supplementation with both, SBS (^¶^Sigma Aldrich (Saint Louis, MO, USA); S9000-500G; Lot # SLBT8383; PCode:102004764) and zearalenone hydrolase ZenA (commercial name: ZEN*zyme*^®^, BIOMIN Holding GmbH, Tulln, Austria; batch ECZ025 (161 U/g)) (−, without supplements; +, with both supplements) as main factors CONT and SUP, respectively. The respective diets were mixed with 50% control maize or with 50% *Fusarium*-toxin contaminated maize in absence (−) or presence (**+**) of 0.62 g (100 U) ZEN*zyme*^®^/kg and 2.5 g SBS/kg (Table 1). Both, CON maize and FUS maize, were cultivated at the experimental station of the Friedrich-Loeffler-Institut in Braunschweig, Germany, under similar agrotechnical conditions. FUS maize was manually inoculated with spores of *Fusarium graminearum* as described by Paulick et al. (2015a) to induce subsequent toxin formation.

**Table 1:** Composition of experimental diets (g/kg as fed)

	Experimental diets			
	CON−	CON+	FUS−	FUS+
**Components**				
Barley	260.00	259.84	260.00	259.84
Control maize (CON)	500.00	499.69	0.00	0.00
* Fusarium* toxin contaminated maize (FUS)	0.00	0.00	500.00	499.69
Soybean meal	181.85	181.74	181.85	181.74
Soybean oil	10.00	9.99	10.00	9.99
Premix^a^	10.00	9.99	10.00	9.99
Lysin-HCl	8.00	8.00	8.00	8.00
DL-Methionine	4.50	4.50	4.50	4.50
L-Threonine	3.50	3.50	3.50	3.50
L-Tryptophan	0.50	0.50	0.50	0.50
Calcium carbonate	9.00	8.99	9.00	8.99
Phytase ZY5000^b^	0.15	0.15	0.15	0.15
HCl-insoluble ash^c^	10.00	9.99	10.00	9.99
Sodium chloride	2.50	0.00	2.50	0.00
Sodium metabisulfite^d^ (SBS)	0.00	2.50	0.00	2.50
ZEN*zyme*^®e^	0.00	0.62	0.00	0.62
**Analyzed composition**				
Deoxynivalenol (DON) (mg/kg), *n* = 5	0.04 ± 0.02	0.03 ± 0.03	2.57 ± 0.26	2.04 ± 0.22
Zearalenone (ZEN) (mg/kg), *n* = 5	< 0.004	< 0.004	0.24 ± 0.06	0.24 ± 0.06
Dry matter (%)	89.6	89.6	90.4	90.3
Crude protein	214.4	216.6	214.0	211.8
Crude fat	32.3	32.2	32.1	33.2
Crude ash	57.3	55.1	54.5	56.3
Crude fiber	45.6	39.3	45.6	39.2

^a^Provided per kg diet: Ca, 1.0 g; P, 1.4 g; Na, 0.9 g; Mg, 0.1 g; vitamin A, 8000 IU; vitamin D_3_, 1000 IU; vitamin E, 50 mg; vitamin B_1_, 1 mg; vitamin B_2_, 3 mg; vitamin B_6_, 2 mg; vitamin B_12_, 20 μg; vitamin K_3_, 2 mg; vitamin C, 50 mg; niacin, 12.5 mg; Ca-pantothenate, 7.5 mg; folic acid, 0.75 mg; biotine, 50 μg; choline chloride, 125 mg; Fe, 75 mg; Cu, 15 mg; Mn, 40 mg; Zn, 50 mg; I,1.0 mg; Se, 0.2 mg; Na_2_SeO_3_, 0.2 mg; Ca(IO_3_)_2_, 1 mg; Mn(II)-O, 40 mg; ZnO, 50 mg; Fe(II)-sulphate-monohydrate, 75 mg; Cu(II)-sulphate-pentahydrate, 15 mg

^b^ZY Phytase 5000 (LOHMANN ANIMAL HEALTH GmbH & Co., Cuxhaven, Germany) with a declared phytase activity (EC 3.1.3.26) of 5000 FYT/g

^c^ > 97% SiO_2_ (Sipernat^®^ 22S, Evonic Industries, Hanau-Wolfgang, Germany)

^d^Sigma Aldrich (Saint Louis, MO, USA); S9000-500G; Lot # SLBT8383; PCode:102,004,764

^e^BIOMIN Holding GmbH (Tulln, Austria), batch ECZ025 (161 U/g), 0.62 g/kg are equivalent to 100 U/kg

Feed additives including ZENzyme and SBS were premixed together with a small amount of barley using a premixer with a 5-kg capacity before being further mixed in the final batch mixer.

DON and ZEN concentrations in the feed of group FUS− as the negative control group were targeted at approximately 3 mg DON/kg and 0.3 mg ZEN/kg and were therefore approximately 3 times higher than the corresponding guidance values for critical diet concentrations of 0.9 mg DON/kg and 0.1 mg ZEN/kg feed, respectively (European Commission 2006). Therefore, anorectic and hyperestrogenic effects were expected at these toxin levels as a precondition to demonstrate putative counteracting effects of the supplements tested with group FUS+. Moreover, to reliably detect mycotoxin residues in physiological specimen indicative for systemic mycotoxin absorption as a prerequisite to investigate the efficacy of supplements, specifically, dietary DON and ZEN levels, markedly higher than the guidance values, are required (Dänicke and Brezina 2013; Dänicke and Winkler 2015). The SBS dose of 2.5 g/kg feed was based on a literature compilation (Dänicke et al. 2012). Authors concluded that approximately 900 mg SBS/mg DON at moisture contents ≥ 15% and a minimum treatment period of 21–28 days would be required to effectively reduce DON concentrations.

### Feeding experiment

For the feeding trial, 80 female weaned crossbred piglets (Bundeshybridzuchtprogramm BHZP, ((German Landrace × German Large White) × Piétrain)), weighing 6.02 ± 0.88 kg, were assigned to four feeding groups (*n* = 20 each) and kept in 20 slatted floor pens (*n* = 5 replications per treatment). In the adaptation period, the weaned piglets were fed ad libitum with the non-supplemented and non-contaminated control (CON −) feed for 7 days. The piglets had free access to water and diets were fed ad libitum.

Live weight and feed intake (per pen) were recorded weekly during the 5-week lasting feeding trial.

Piglets were assessed weekly during the trial period and immediately after slaughter: tail and both ears were scored according to specifications of the German Pig-Scoring Key (Bönisch et al. 2017) detail level 2. On the tail and ears, it was recorded whether the skin was injured, whether there was necrosis or loss, and additionally on the tail whether there was ring constriction or swelling. In the assessment of the tail, the parameter skin injury was recorded in three levels, with 0 indicating no injury, 1 indicating a small injury (maximum as large as diameter of the tail at the respective location) and 2 indicating a large injury (larger than diameter of the tail at the respective location). Similarly, the parameter loss was recorded in three levels, where 0 corresponded to no loss, 1 to a partial loss, and 2 to a full loss of the tail. The other parameters on the tail, as well as all parameters on ear, were recorded in two levels, where 0 indicating that the corresponding parameter did not occur, and 1 that the parameter occurred. The claws were individually assessed divided into sub-areas based on the scheme of Ziron (2014). The claw as a whole, the sole, the pads, the coronet band, and the claw wall were assessed for lesions, and it was also recorded whether a panaritium had occurred. All parameters for claws were recorded in three levels, with level 0 indicating no lesion, level 1 a slight lesion, and level 2 a strong lesion. The mammary complex was assessed for injuries, lesions, or abnormalities, including swelling and reddening, in two levels, with level 0 indicating no lesion and level 1 the presence of a lesion. The vulva was assessed in two levels. Level 0 corresponded to no swelling or injury, level 1 to swelling and injury. As quantitative measures, the height and width of the vulva were determined in addition to vulva scoring, using a digital calliper.

For further data evaluation, the scores for the complexes tail, ears, vulva, mammary complex, and claws were cumulated whereby the resulting scores of 6, 8, 1, 1, and 24, respectively, would represent the worst evaluation.

After final live weight recording, piglets were electrically stunned and exsanguinated. Blood samples were collected via venipuncture of the neck vessels just before exsanguination.

Organs were quickly dissected from the carcasses, recording organ weights including uterus and ovaries. Furthermore, urine for mycotoxin residue analysis was collected by urinary bladder puncture. Finally, feces from the rectum was collected for similar analytical purposes.

Feed samples were collected weekly (*n* = 5) and analyzed individually for recording the time course of mycotoxin concentration over the experimental period of 5 weeks while feed samples were pooled over the entire period for analyzing crude nutrients and mycotoxins.

The experiment was performed according to the European Commission regulations concerning the protection of experimental animals and the guidelines of the Regional Council of Braunschweig, Lower Saxony, Germany (file number 33.19-42,502-04-16/2325).

### Analysis

#### Crude nutrients in feed

Pooled feed samples were ground to pass a 0.5 mm screen and analyzed for crude nutrients according to the methods of the VDLUFA (Verband Deutscher Landwirtschaftlicher Untersuchungs- und Forschungsanstalten 1976). In particular, the following methods were used: No. 3.1 for dry matter (DM), No. 4.1.1 for Kjeldahl Nitrogen, No. 5.1.1 for ether extract, and No. 6.1.1 for crude fiber.

#### Hematology

For hematology, fresh EDTA blood samples (Sarstedt, Nümbrecht, Germany) were analyzed using an automatic hematology analyzer (Celltac-α, MEK 6450, Nihon Kohden Corporation, Tokyo, Japan) which enables determining the following parameters and indices derived therefrom: white blood cells (WBC), red blood cells (RBC), hemoglobin (HGB), hematocrit (HCT), mean corpuscular volume (MCV), mean corpuscular hemoglobin (MCH), mean corpuscular hemoglobin concentration (MCHC), red cell distribution width (RDW), platelet count (PLT), plateletcrit (PCT), mean platelet volume (MPV), and platelet distribution width (PDW).

For differential white blood cell count, three blood smears per animal were prepared, stained according to Pappenheim, and 200 cells counted manually per slide by light microscopy (Nikon Eclipse E200, Nikon Europe b.v., Badhoevedorp, The Netherlands). Lymphocytes, reticulocytes, banded and segmented neutrophils, basophiles, eosinophils, and monocytes were expressed as total counts by multiplying their corresponding proportions with total WBC.

#### Clinical chemistry

Clinical-chemical traits were analyzed in blood serum which was prepared from supernatants of clotted blood samples centrifuged at 2123 g for 15 min at 15 °C. The IndikoPlus (Thermo Fischer Diagnostics GmbH, Henningsdorf, Germany) was used to determine the following parameters photometrically: phosphorus, calcium, triglycerides, cholesterol, albumin, total bilirubin, direct bilirubin, urea, alkaline phosphatase (**ALP**), creatinine, gamma-glutamyl-transferase (**GGT**), glutamate dehydrogenase (**GLDH**), alanine-amino-transferase (**ALT**), aspartate-amino-transferase (**AST**), total protein, and glucose. Chloride, potassium, and sodium were detected by ion-selective electrodes.

#### Creatinine content in urine and mycotoxins in feed, urine, blood, and feces

The creatinine content in urine samples was measured by LC–MS/MS after diluting the samples with water 1:5000 according to Warth et al. (2012). For extraction of mycotoxins and metabolites in urine, the samples were diluted with water to a concentration of 0.2 mM creatinine, incubated with β-glucuronidase from *E. coli* type IX-A (Sigma-Aldrich, Saint Louis, MO, USA) overnight at 37 °C. The reaction was stopped with methanol/formic acid (99/1 v/v), the samples were centrifuged, and subjected to HPLC–MS/MS analysis as described below.

Freeze-dried feces samples were homogenized and aliquoted into 500 mg portions. They were extracted with 5 mL of acetonitrile/water (50/50, v/v) on a GFL rotary shaker (type 3017, Burgwedel, Germany) three times (90/30/30 min). The extract was analyzed by HPLC–MS/MS.

Plasma samples were incubated with β-glucuronidase from *E. coli* type IX-A (Sigma-Aldrich, Saint Louis, USA) overnight at 37 °C. Proteins were precipitated using methanol/formic acid (99/1 v/v) and after centrifugation, the samples were subjected to HPLC–MS/MS analysis as described below.

For the analysis of feed samples, feed was extracted as described in Schwartz-Zimmermann et al. (2014a).

All extracts were analyzed for DON, ZEN, and their metabolites by HPLC–MS/MS as described in Schwartz-Zimmermann et al. (2014b) and Binder et al. (2017) with minor modifications. All compounds were separated on a Kinetex EVO C18 2.6 µm 150 × 2.1 mm (Phenomenex, Aschaffenburg, Germany) using a gradient elution. Mobile phase A contained water/acetonitrile (95/5 v/v) and mobile phase B consisted of acetonitrile/water (95/5 v/v). Both mobile phases contained 0.1% acetic acid. The flow rate was 0.65 mL/min, the temperature in the column compartment was set to 30 °C, and the injection volume was set to 3 µL. Total run time was 7.5 min. In addition to analytes described in these publications, HZEN and DHZEN were measured using the following *m/z* transitions: 335/149 Da, collision energy − 34 V; and 291/149 Da, collision energy − 25 V. These analyses were performed on an Agilent Infinity II 1290 UHPLC system coupled to an AB Sciex QTRAP 6500 + Triple Quad mass spectrometer equipped with a Turbo V^®^ ion source (SB Sciex, Foster City, CA, USA).

Limits of detection (LOD) and limits of quantification (LOQ) for the various matrices are presented in Table 2. LOD and LOQ were determined using the signal-to-noise approach. In addition, accuracy and precision criteria had to be fulfilled for LOQ.

**Table 2:** Limit of detection (LOD) and quantification (LOQ) of deoxynivalenol (DON), zearalenone (ZEN), and their metabolites in feed and various physiological matrices

	Feed (ng/g)		Plasma (ng/ml)		Urine (ng/ml)		Feces (ng/g)
	LOD	LOQ	LOD	LOQ	LOD	LOQ	LOQ
ZEN	7.6	38	0.15	0.45	0.20	1.00	27.00
α-ZEL	7.6	38	0.30	0.90	0.40	2.00	27.00
β-ZEL	7.6	38	0.60	1.80	0.40	2.00	27.00
HZEN	76	380	0.15	0.45	0.50	2.50	10.80
DHZEN	7.6	38	0.60	1.80	0.50	2.50	108.00
DON	19	95	0.75	2.25	0.50	2.50	27.00
DOM	19	95	3.00	9.00	0.50	2.50	270.00
DONS1	19	95	1.50	4.50	0.25	1.30	27.00
DONS2	19	95	0.75	2.25	0.25	1.30	27.00
DONS3	19	95	0.75	2.25	0.25	1.30	27.00

*ZEL* zearalenol, *HZEN* hydrolyzed ZEN, *DHZEN* decarboxylated hydrolyzed ZEN, *DOM* de-epoxy-DON, *DONS1, 2, 3* DON sulfonates 1,2, 3

### Calculation and statistics

Results were evaluated using Statistica 13.0 (StatSoft, Inc. 2014, Tulsa, Oklahoma, USA) and SAS Enterprise Guide 6.1 (SAS Institute 2013, Cary, NC, USA).

Data were generally evaluated according to a complete 2 × 2-factorial design with *Fusarium* toxin contamination (CON, background contaminated control feed; FUS, *Fusarium* toxin contaminated feed) and supplementation with SBSand ZenA ( −, without supplements; +, with both supplements) as main factors CONT and SUP, respectively, and their interactions using analysis of variance (ANOVA) or proc mixed, depending on further model extensions. When parameters were recorded repeatedly during the experiment, the fixed factor time (TIME) and all related interactions were additionally included in the model. Similarity between subjects was considered through “Repeated” or “Random” statements. Depending on distribution of residues, statistics was performed on untransformed or rank-transformed data (proc rank). Data are presented as Lsmeans or as means and pooled standard error of means, or as box plots (medians, 25th and 75th percentiles, minimum and maximum) depending on evaluation strategy for the 4 experimental groups representing the interactions between SUP and CONT. Multiple comparisons were performed by Tukey’s test only if these interactions were significant.

Before performing statistics on mycotoxin residues in plasma, urine or feces concentrations were set to zero, when they were lower than LOQ.

## Results

### Mycotoxins in feed

**Fig. 1:**
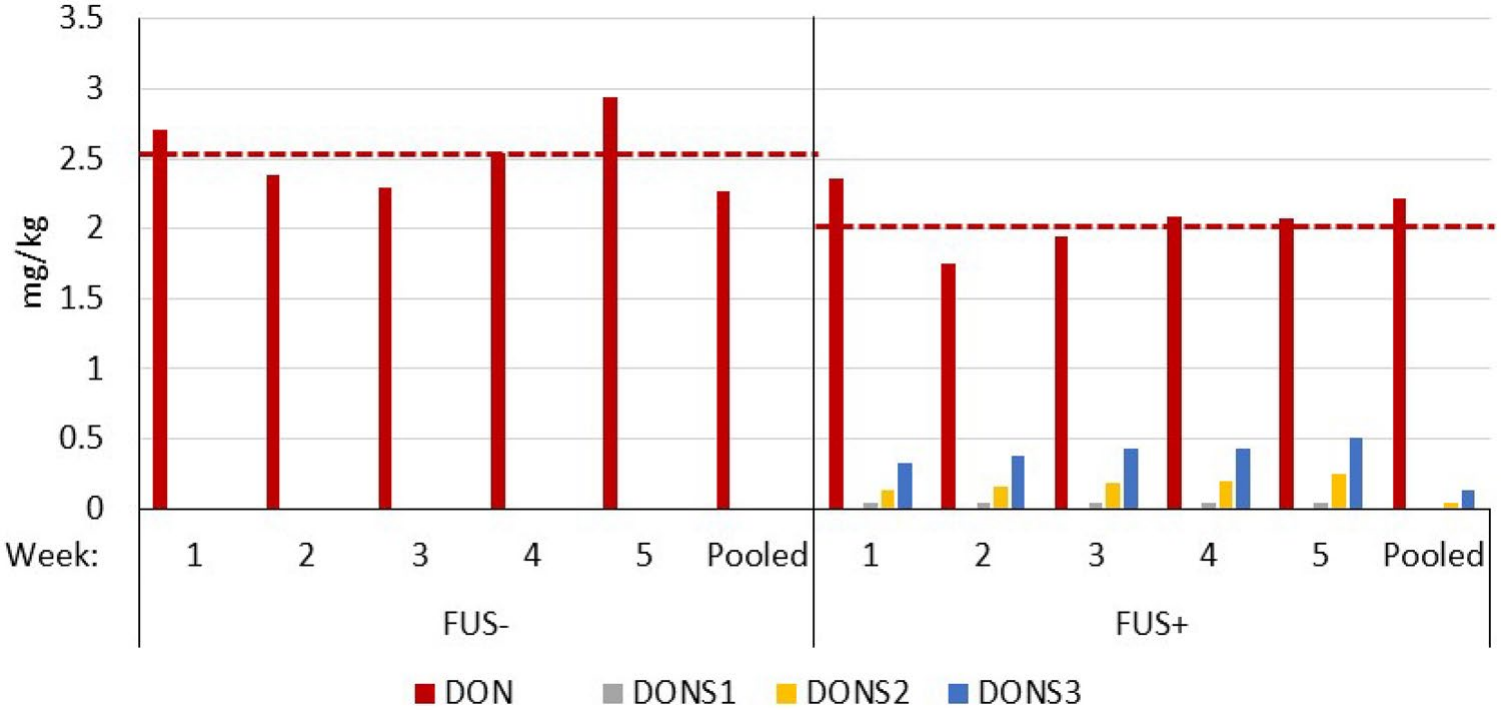
Time course of deoxynivalenol (DON) and DON-sulfonate 1, 2, and 3 (DONS1, DONS2, DONS3) concentrations in diets (mg/kg as fed) containing *Fusarium* toxin contaminated maize (FUS) in the absence or presence of supplements (− / +) compared to a feed sample pooled over the whole experiment. Dashed horizontal lines represent the mean DON concentrations (*n* = 5) and were significantly different between FUS− and FUS+ (*p* = 0.008). Concentrations of de-epoxy-DON (DOM) were lower than LOQ

DON concentration in diet FUS− varied only slightly and undirectedly between 2.2 and 2.94 mg/kg (Fig. 1). The mean value of 2.57 mg DON/kg comprising 5 weekly samples analyzed individually matched that of 2.27 mg DON/kg determined for the pooled sample quite reasonable. The supplemented FUS+ diet contained approximately 20% less DON compared to FUS− diet on average. The highest DON concentration of 2.36 mg/kg was found at the beginning of the experiment, while the lowest one of 1.75 mg/kg was detected in the 2nd week. DON concentrations of the following 3 samples were compared approximately to the mean value of 2.04 mg/kg. DON sulfonates were present already at the beginning of the experiment and DONS2 and DONS3 appeared to increase in the course of the experiment up to approximately 0.25 and 0.5 mg/kg, respectively.

**Fig. 2:**
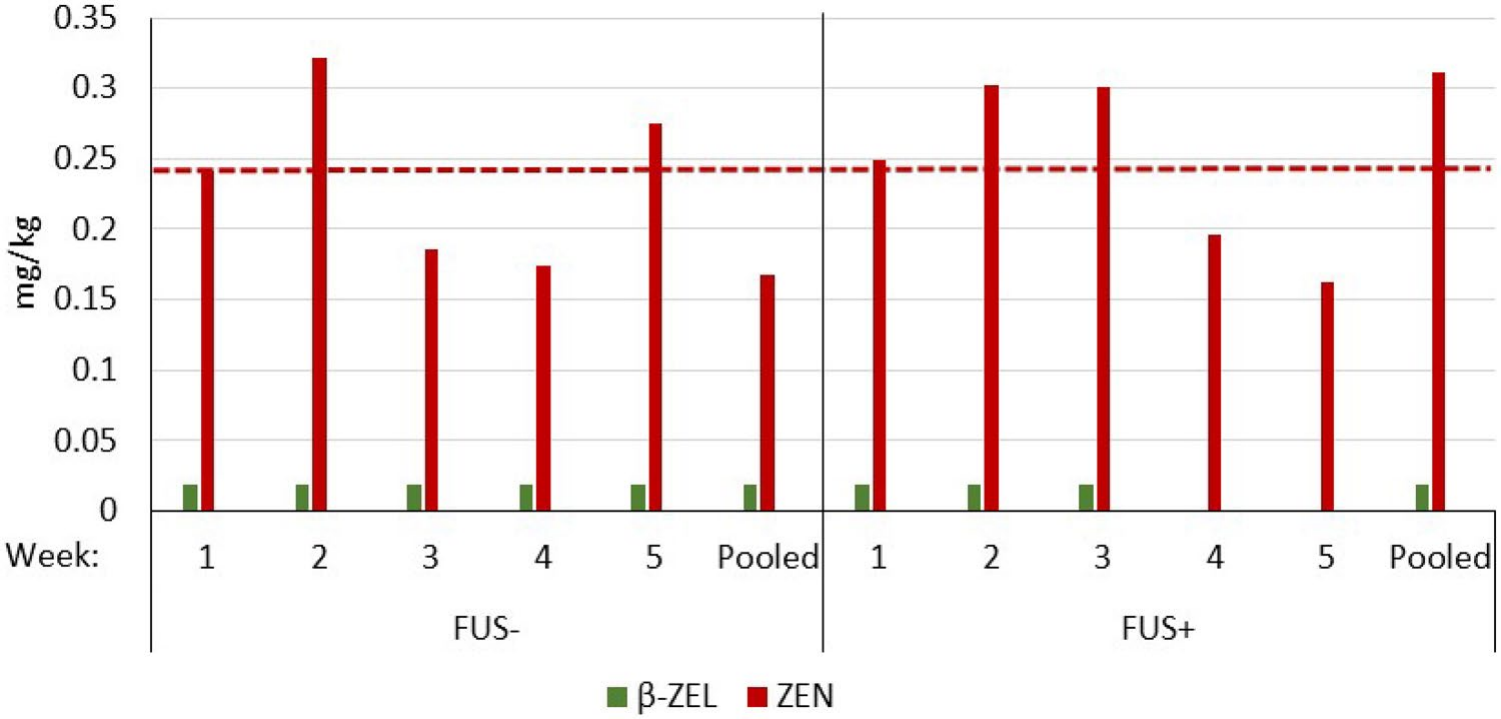
Time course of zearalenone (ZEN) and β-zearalenol (β-ZEL) concentrations in diets (mg/kg as fed) containing *Fusarium* toxin contaminated maize (FUS) in the absence or presence of supplements (− / +) compared to a feed sample pooled over the whole experiment. Dashed horizontal lines represent the mean ZEN concentrations (*n* = 5) and were not significantly different between FUS− and FUS+ (*p* > 0.05). Concentrations of α-ZEL, hydrolyzed ZEN (HZEN), and decarboxylated hydrolyzed ZEN (DHZEN) were lower than the corresponding LOQs

ZEN concentrations of both the FUS− and FUS+ diet varied undirectedly between 0.17 and 0.32 mg/kg, and 0.16 and 0.3 mg/kg, respectively with a similar mean value of 0.24 mg/kg (Fig. 2). ZEN concentrations of the pooled samples fitted within this overall variation. Amongst the ZEN metabolites, only β-ZEL was detected irregularly in trace concentrations.

### Performance of piglets

One piglet of the group CON+ had to be excluded during the first week of the experiment for welfare reasons caused by poor performance probably associated with the onset of the study but not associated to the dietary treatment.

Supplements increased feed intake and live weight gain significantly irrespective of diet type by 15% and 28%, respectively (Table 3). This effect occurred at a significantly lower level in FUS-fed groups and amounted to − 14% and − 15% for feed intake and live weight gain, respectively, compared to CON groups. Feed to gain ratio was improved by 9% independently of diet type.

**Table 3:** Performance parameters of piglets fed diets containing contaminated (FUS) or uncontaminated (CON) maize in absence (−) or presence (+) of mycotoxin inactivating feed additives for 5 weeks (LS-Means, *n* = 5)

	Experimental diet				*p*-values			
	CON**− **	CON+	FUS**− **	FUS+	CONT	SUP	CONT×SUP	PSEM
Live weight gain (g/d)	323	427	285	349	**< 0.001**	**0.003**	0.259	16
Feed intake (g/d)	538	634	474	533	**0.002**	**0.004**	0.424	23
Feed to gain (kg/kg)	1.66	1.49	1.67	1.53	0.314	**0.005**	0.238	0.04

*p*-values <0.05 are emphasised in bold

*LS-Means*, least square means, *PSEM* pooled standard error of means, *CONT Fusarium* toxin contamination of feed, *CON* uncontaminated control feed, *FUS Fusarium* toxin contaminated feed, *SUP* supplementation of feed, − without supplements, + with ZEN hydrolase ZenA plus sodium metabisulfite

### Scoring of various trait complexes of piglets

**Fig. 3:**
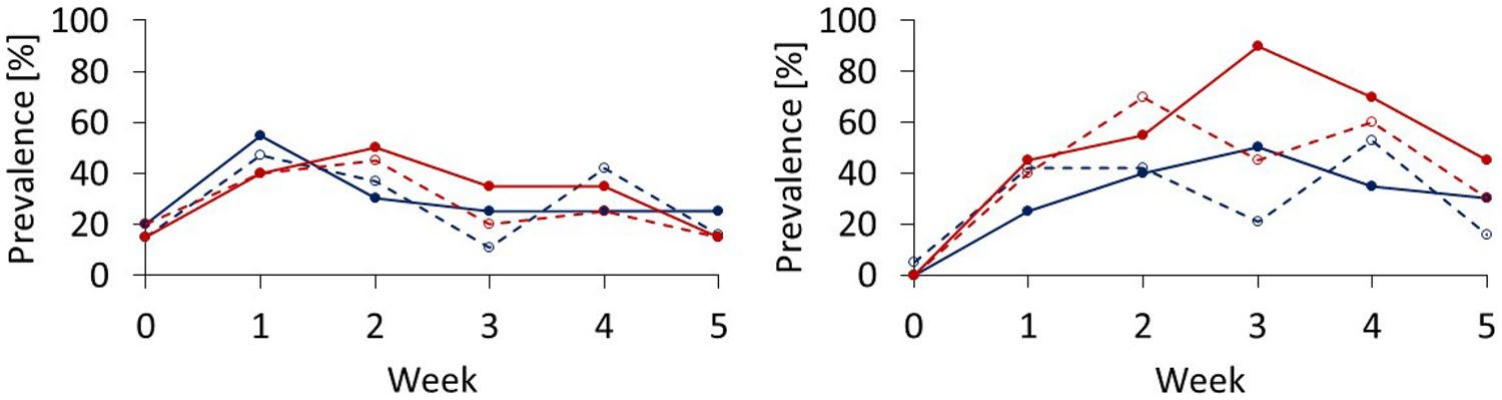
Prevalence (% of affected pigs) of tail injuries (left) and swelling and/or reddening of the mammary complexes (right) in the course of the experiment CON−,  , CON+,  , FUS−,  , FUS+,

  Generally, weekly gross inspection of tail, ears, vulva, and claws revealed no significant effects of *Fusarium* toxin contamination and of supplements (Table 4). The overall maximum mean value accounted for 6, 8, 2, and 3% of the total scores of 6, 8, 1, and 24, respectively. While vulva was completely inconspicuous between the groups and weeks of inspection (data not shown), a closer look to the particular traits of the underlying scores of ears and tails revealed nearly exclusively injuries with prevalence decreasing in the course of the experiment from 48 to 9%, and 46 to 18%, respectively (Fig. 3), but only occasionally necrosis in 1–2 pigs per group distributed irregularly over groups and weeks. The evolution of the total claw score was based mainly on claw wall lesions with a mean prevalence of 4 and 10% for the hind and front claws, respectively, whereby a maximum of 5 pigs were affected irregularly and independent of group (data not shown). Besides claw lesions, lesions of the coronary bands contributed to the total claw score but again independently of group and week with a mean prevalence of 1 and 5% for hind and front claws corresponding to a maximum of 3 pigs affected (data not shown). Sole and pad lesions as well as panaritium were diagnosed only sporadically and irregularly in individual animals irrespective of group affiliation (data not shown).

**Table 4:** Gross lesion scores for various trait complexes of piglets fed diets with *Fusarium* contaminated (FUS) or uncontaminated (CON) maize in absence ( −) or presence ( +) of mycotoxin inactivating feed additives (*n*(CON −, FUS−, FUS +) = 20; *n*(CON +) = 19)

	Tail	Ears	Mammary complex	Claws
Experimental diets				
CON**− **	0.31	0.58	0.30	0.42
CON+	0.28	0.52	0.32	0.61
FUS**− **	0.34	0.62	0.52	0.45
FUS+	0.28	0.67	0.41	0.45
*p*-values				
CONT	0.760	0.342	**0.012**	0.517
SUP	0.466	0.995	0.474	0.329
TIME	**< 0.001**	** < 0.001**	**< 0.001**	**< 0.001**
CONT×SUP	0.794	0.606	0.258	0.329
CONT×TIME	0.362	0.981	0.088	0.527
SUP×TIME	0.834	0.818	**0.002**	0.979
CONT×SUP×TIME	0.752	0.207	0.459	0.234
PSEM	0.03	0.05	0.03	0.05

*p*-values <0.05 are emphasised in bold

Values are presented as means

*PSEM* pooled standard error of means, *CONT Fusarium* toxin contamination of feed, *CON* uncontaminated control feed, *FUS Fusarium* toxin contaminated feed, *SUP* supplementation of feed, − without supplements, + with ZEN hydrolase ZenA plus sodium metabisulfite

A significant effect of the *Fusarium* toxin contamination was observed for the mammary complex and the maximum score of 1 was reached by approximately half of the observations (~ 52%) in group FUS− while a lower prevalence was observed in group FUS+ and both control groups (Fig. 3, Table 4).

### Morphometry of vulvae

In addition to the semi-quantitative scoring of vulvae and mammary complexes, the height and width of vulvae were recorded as quantitative continuous features (Table 5). Both measures increased significantly with time. The time-related increase in vulva width was more pronounced in group FUS− compared to group CON−, while the opposite was observed for groups FUS+ and CON+ giving rise to the significant interactions between *Fusarium* toxin contamination of feed (CONT) and supplements (SUP).

**Table 5:** Height and width (mm) of vulva of piglets fed diets with Fusarium contaminated (FUS) or uncontaminated (CON) maize in absence (**−**) or presence (+) of SBS and ZEN hydrolase ZenA (LS-Means, n(CON**−**, FUS**−**, FUS+) = 20; *n*(CON+) = 19)

	Height	Width
Experimental diet		
CON**− **	16.6	10.7
CON+	16.1	11.4
FUS**− **	16.4	11.4
FUS+	17.2	10.8
*p*-values		
CONT	0.288	0.899
SUP	0.767	0.945
CONT× SUP	0.133	**0.013**
TIME	** < 0.01**	** < 0.01**
TIME× CONT	0.663	0.454
TIME× SUP	0.101	0.722
TIME× CONT×SUP	0.01	0.318
PSEM	0.23	0.18

*p*-values <0.05 are emphasised in bold

*LS-Means* least square means, *PSEM* pooled standard error of means, *CONT Fusarium* toxin contamination of feed, *CON* uncontaminated control feed, *FUS Fusarium* toxin contaminated feed, *SUP* supplementation of feed, − without supplements, + with ZEN hydrolase ZenA plus sodium metabisulfite

### Clinical chemistry

Triglycerides, cholesterol, and glucose were analyzed as indicators for energy and lipid metabolism (Table 6). From these traits, only cholesterol was influenced by treatments in an interactive manner. Supplements significantly increased the cholesterol levels only in group CON+, while no effect of additives was noticed for group FUS+ compared to its unsupplemented counterpart. Total protein, urea, and creatinine remained unaltered while albumin concentration was slightly reduced in FUS-fed piglets by 5% and significantly increased by supplements by 13% irrespective of diet type. Out of biochemical traits mainly indicative for liver integrity, GLDH, bilirubin fractions, and ALT were influenced by treatments, while ALP, GGT, and AST remained uninfluenced. Though total and direct bilirubin were significantly increased by the supplements with CON as diet type, these fractions remained unaltered when supplements were added to the FUS diet. Whereas the supplements significantly decreased the activity of ALT independent of diet type, the GLDH levels were increased at the same time albeit to a larger extent in group CON+ when compared to group FUS+. Amongst the electrolytes, phosphorus, chloride, potassium, sodium, and calcium, only the latter was influenced by treatments. Calcium concentrations were significantly increased by 6% as a consequence of the addition of supplements irrespective of diet type.

**Table 6:** Clinical-chemical parameters of piglets fed diets containing contaminated (FUS) or uncontaminated (CON) maize in absence (−) or presence (+) of mycotoxin inactivating feed additives for five weeks (LS-Means, *n*(CON**−**, FUS**−**, FUS+) = 20; *n*(CON+) = 19)

	Experimental diet				*p*-values			
	CON**− **	CON+	FUS**− **	FUS+	CONT	SUP	CONT×SUP	PSEM
Triglycerides (mmol/l)	0.45	0.49	0.46	0.45	0.635	0.674	0.352	0.002
Cholesterol (mmol/l)	1.80^b^	2.09^a^	1.71^b^	1.68^b^	**0.001**	0.083	**0.035**	0.004
Albumin (g/l)	29.8	32.4	27.4	32.02	0.088	**< 0.001**	0.216	0.048
Bilirubin total (µmol/l)	1.62^b^	2.22^a^	1.96^ab^	1.97^ab^	0.692	**0.010**	**0.013**	0.007
Bilirubin direct (µmol/l)^a^	0.17^b^	0.83^a^	0.58^ab^	0.49^ab^	0.767	**0.027**	**0.012**	0.008
Urea (mmol/l)	1.42	1.15	1.61	1.48	0.330	0.457	0.795	0.016
Creatinine (µmol/l)	70.4	68.7	72.3	70.6	0.496	0.547	0.997	0.169
Total protein (g/l)	50.2	49.0	48.0	48.4	0.357	0.784	0.581	0.089
Glucose (mmol/l)	7.98	7.96	7.69	7.95	0.573	0.655	0.603	0.016
ALP (µkat/l)^a^	4.24	4.30	4.41	4.98	0.109	0.232	0.348	0.016
GGT (µkat/l)	0.59	0.66	0.66	0.64	0.483	0.388	0.193	0.002
ALT (µkat/l)	0.85	0.75	0.90	0.80	0.164	**0.005**	0.891	0.002
AST (µkat/l)	0.78	0.93	0.94	0.94	0.130	0.158	0.138	0.003
GLDH (µkat/l)	0.05^b^	0.08^a^	0.06^ab^	0.07^ab^	0.976	**0.001**	**0.042**	0.0004
Phosphorus (mmol/l)	3.08	3.07	2.79	3.12	0.252	0.121	0.106	0.006
Chloride (mmol/l)	108.5	106.6	107.6	107.4	0.921	0.066	0.124	0.033
Potassium (mmol/l)	10.9	10.7	10.2	10.4	0.159	0.938	0.467	0.020
Sodium (mmol/l)	139.5	138.2	139.2	139.1	0.613	0.263	0.318	0.038
Calcium (mmol/l)	2.81	3.00	2.84	2.98	0.932	**0.035**	0.731	*0.005*

*p*-values <0.05 are emphasised in bold

*LS-Means* least square means, *PSEM* pooled standard error of means, *CONT* Fusarium toxin contamination of feed, *CON* uncontaminated control feed, *FUS* Fusarium toxin contaminated feed, *SUP* supplementation of feed, −  without supplements, + with ZEN hydrolase ZenA plus sodium metabisulfite, *ALP* alkaline phosphatase, *GGT* Gamma-glutamyl transferase, *ALT* alanine-aminotransferase, *AST* aspartate-amino-transferase, *GLDH* glutamate-dehydrogenase

^a^ANOVA was performed using the ranks. Different superscripts indicate significant differences between treatment groups (*p* < 0.05) in case of significant interactions

### Hematology

White blood cell counts were significantly reduced by approximately 19% by the supplements irrespective of diet type (Table 7). A significant similarly directed diet-independent reduction in banded neutrophil granulocytes was observed as a result of adding the supplements to the diets. Reticulocytes, segmented neutrophil, eosinophil, and basophil granulocytes as well as monocytes remained unaltered. While red blood cell count, hemoglobin content, and hematocrit were not influenced by the treatments, the erythrocyte indices MCV and MCH were significantly increased by 3% when supplemented diets were fed. The mean corpuscular hemoglobin concentration was significantly reduced in FUS-fed groups independently of supplements. Platelet counts were significantly reduced by 18% as a consequence of adding the supplements to both diet types. The other platelet traits plateletcrit, mean corpuscular volume, and platelet distribution width remained inconspicuous.

**Table 7:** White and red hemogram of piglets fed diets containing control (CON) or *Fusarium* toxin contaminated maize (FUS) in the absence (−) or presence (+) of mycotoxin inactivating feed additives for five weeks (LS-means*, *n*(CON−, FUS−, FUS+) = 20; *n*(CON +) = 19)

	Experimental diet				*p*-values			
	CON**− **	CON+	FUS**− **	FUS+	CONT	SUP	CONT×SUP	PSEM
**White hemogram (10**^**3**^**/µl)**								
WBC	25.24	21.96	27.30	20.85	0.758	**0.002**	0.299	0.091
Lymphocytes	16.10	14.82	16.23	14.42	0.608	0.054	0.521	0.058
Banded neutrophils	7.19	5.39	8.21	5.26	0.569	**0.026**	0.461	0.046
Segmented neutrophils^#^	1.51	1.26	2.40	1.38	0.495	0.544	0.919	0.020
Basophiles	0.02	0.06	0.04	0.07	0.468	0.155	0.773	0.001
Eosinophils^#^	0.40	0.41	0.41	0.33	0.739	0.288	0.863	0.004
Monocytes	0.03	0.02	0.02	0.05	0.656	0.676	0.271	0.001
Reticulocytes	0.45	0.36	0.36	0.25	0.173	0.202	0.904	0.005
**Red hemogram**								
RBC (10^6^/µl)	6.92	6.71	6.46	6.49	0.096	0.648	0.565	0.012
HGB (g/dl)	11.44	11.71	10.96	11.10	0.111	0.545	0.847	0.020
HCT (%)	37.55	38.14	35.98	36.51	0.152	0.614	0.978	0.066
MCV (fl)	54.34	57.04	55.63	56.34	0.641	**0.008**	0.115	0.037
MCH (p/g)	16.56	17.51	16.96	17.13	0.936	**0.007**	0.054	0.012
MCHC (g/dl)	30.47	30.71	30.46	30.40	**0.041**	0.249	0.056	0.005
RDW (%)	16.29	15.95	16.61	16.13	0.301	4.170	0.085	0.012
PLT (10^3^/µl)^#^	418.18	357.95	431.35	338.20	0.922	**0.025**	0.624	1.993
PCT (%)	0.24	0.21	0.25	0.21	0.873	0.051	0.993	0.001
MPV (fl)	5.81	5.82	5.82	5.93	0.677	0.645	0.729	0.008
PDW (%)	14.22	14.48	14.31	14.39	0.987	0.321	0.594	0.010

*p*-values <0.05 are emphasised in bold

*LS-Means* least square means, *PSEM* pooled standard error of means, *CONT* *Fusarium* toxin contamination of feed, *CON* uncontaminated control feed, *FUS* *Fusarium* toxin contaminated feed, *SUP* supplementation of feed, – without supplements, +, with *ZEN* hydrolase ZenA plus sodium metabisulfite, *WBC*, white blood cell, *RBC*, red blood cells, *HGB* hemoglobin, *HCT* hematocrit *MCV* mean corpuscular volume, *MCH* mean corpuscular hemoglobin, *MCHC* mean corpuscular hemoglobin concentration, *RDW* red cell distribution width, *PLT* platelet count, *PCT* plateletcrit, *MPV* mean platelet volume, *PDW* platelet distribution width

^a^ANOVA was performed using the ranks

### Organ weights

Body weight at slaughter was significantly lower by 11% in FUS-fed piglets compared to the CON-fed groups while supplements increased body weight by 17% independent of diet type (Table 8). Pancreas weights relative to body weight were significantly increased in piglets fed the FUS diets compared to their CON-fed counterparts while heart and lung weights were significantly reduced by 6 and 12%, respectively, in piglets fed the supplemented diets.

**Table 8:** Live weight and relative organ weights (g/kg live weight) of piglets fed diets with *Fusarium* contaminated (FUS) or uncontaminated (CON) maize in absence (−) or presence (+) of mycotoxin inactivating feed additives (LS-Means*, *n*(CON−, FUS−, FUS+) = 20; n(CON+) = 19)

	Experimental diet				*p*-values			
	CON**− **	CON+	FUS**− **	FUS+	CONT	SUP	CONT×SUP	PSEM
Live weight (kg)	18.28	21.89	16.70	18.98	**0.008**	**0.001**	0.417	0.049
Liver	25.09	24.91	24.92	25.34	0.802	0.812	0.549	0.029
Spleen	2.11	2.00	2.19	2.10	0.366	0.305	0.926	0.006
Pancreas	2.08	2.18	2.34	2.35	**0.013**	0.553	0.564	0.005
Kidneys	4.56	4.53	4.53	4.52	0.877	0.826	0.933	0.006
Heart	5.19	4.93	5.49	5.15	0.065	**0.033**	0.774	0.008
Lung	16.47	14.01	15.99	14.60	0.947	**0.012**	0.479	0.044

*p*-values <0.05 are emphasised in bold

*LS-Means* least square means, *PSEM* pooled standard error of means, *CONT Fusarium* toxin contamination of feed, *CON* uncontaminated control feed, *FUS Fusarium* toxin contaminated feed, *SUP* supplementation of feed, − without supplements, + with ZEN hydrolase ZenA plus sodium metabisulfite

**Fig. 4:**
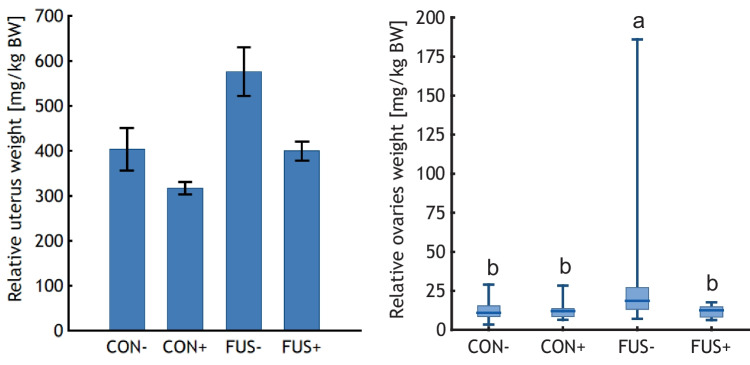
Uterus and ovaries weights relative to live weight of piglets fed diets containing control (**CON**) or *Fusarium* toxin contaminated maize (**FUS**) in the absence or presence of supplements (**−** /** +**) for 5 weeks. Relative uterus weights are presented as LS-Means with standard errors as whiskers (p_FUS_ = 0.001, p_SUP_ = 0.001, p_FUS*SUP_ = 0.244). Ovary weights are shown as boxplots with dashes as medians and minimum and maximum values as whiskers (median ± min/max) (p_FUS_ = 0.041, p_SUP_ = 0.016, p_FUS*SUP_ = 0.027). Boxes indicate the 25th and 75th percentile. ANOVA and post hoc Tukey’s test were performed using ranks. Different lower-case letters indicate significant differences between treatment groups (*p* < 0.05)

Relative uterus weights (Fig. 4) were significantly increased in FUS-fed piglets compared to their CON-fed counterparts and supplements resulted in a significant decrease when the main effect of supplementation was considered. Although the interaction between *Fusarium* toxin contamination and supplementation was insignificant, the relative uterus weight of group FUS− was approximately 1.5-fold heavier compared to the mean value of the 3 other feeding groups. Similarly, the relative ovary weights (Fig. 4) reflected the relationships amongst the treatment groups as described for the relative uterus weights. Here, the interaction between *Fusarium* toxin contamination and supplementation proved to be significant.

### Mycotoxin residues in blood plasma, urine, and feces

**Fig. 5:**
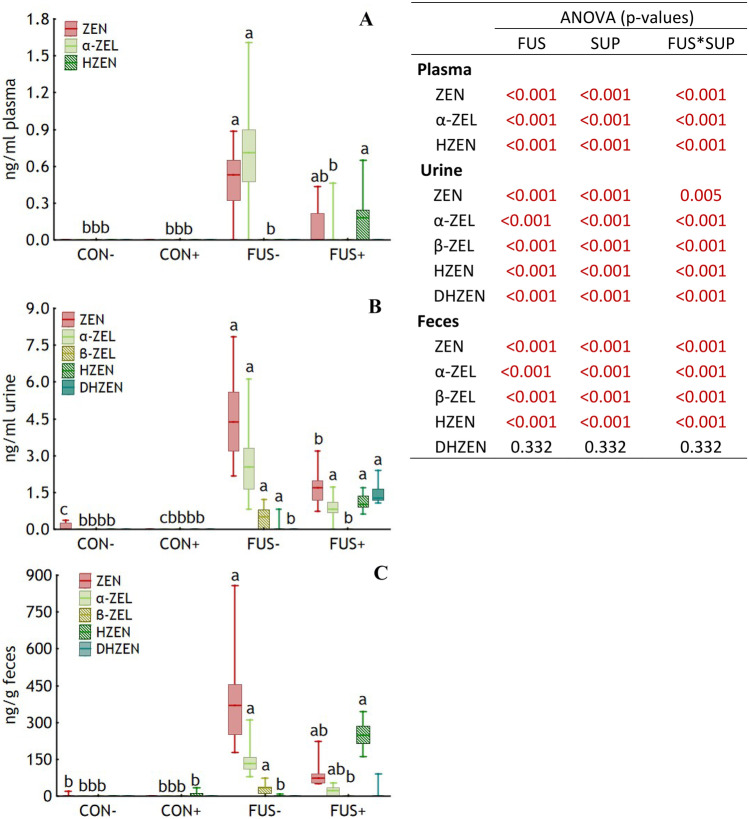
Concentrations of zearalenone (ZEN) and its metabolites in plasma (**A**), urine (**B**), and feces (**C**) of piglets fed diets containing control (**CON**) or *Fusarium* toxin contaminated maize (**FUS**) in the absence or presence of mycotoxin inactivating feed additives (− / **+**) for 5 weeks. Boxes indicate the 25th and 75th percentile, dashes the medians and whiskers the minimum and maximum values. ANOVA and post hoc Tukey’s test for significant interactions were performed using ranks. Different lower-case letters indicate significant differences between treatment groups (*p* < 0.05). α-ZEL, α-zearalenol (ZEL); β-ZEL; HZEN, hydrolyzed ZEN; DHZEN, decarboxylated hydrolyzed ZEN. *n* = 20, 20, and 17 for urine, plasma, and feces of group CON−, respectively; *n* = 18, 19, and 16 for urine, plasma, and feces of group CON+, respectively; *n* = 16, 20, and 12 for urine, plasma, and feces of group FUS−, respectively; *n* = 20, 20, and 17 for urine, plasma, and feces of group FUS+, respectively

Only trace concentrations of ZEN were occasionally detected in urine and feces of group CON−, and of HZEN in feces of group CON+ (Fig. 5). Relevant ZEN residue concentrations were detected in all analyzed specimens of both groups fed the FUS diets, but with significant differences in level and pattern of metabolites. ZEN and α-ZEL were the predominant metabolites in group FUS− in all 3 specimen. In group FUS+, ZEN and α-ZEL concentrations were lower compared to group FUS− and HZEN and DHZEN were additionally detected. Except for DHZEN, for all metabolites and specimens significant interactions between FUS and SUP were observed pointing at the mentioned differences in levels of individual metabolites between CON groups, and groups FUS−and FUS+.

**Fig. 6:**
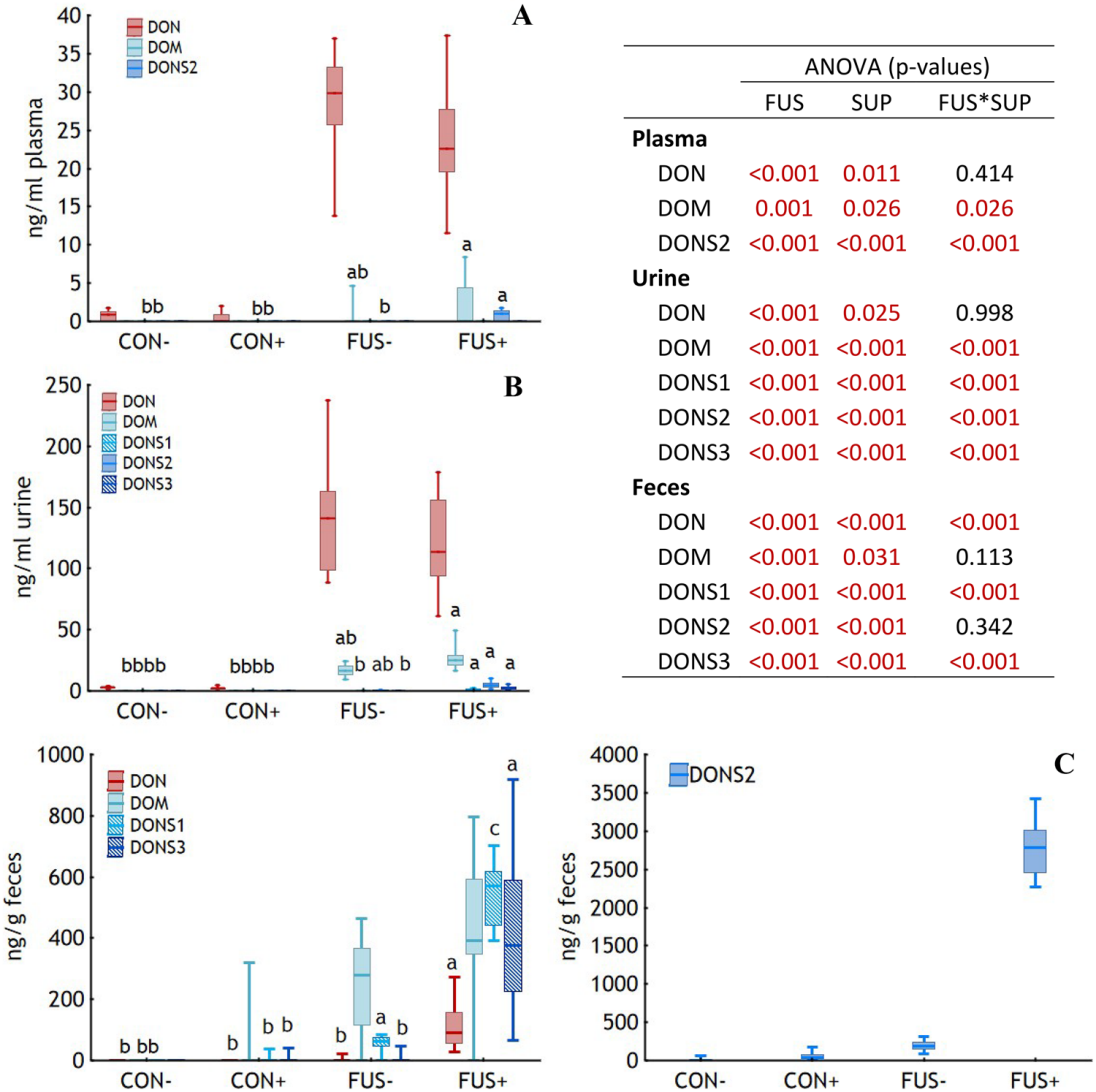
Concentrations of deoxynivalenol (**DON**) and its metabolites in plasma (**A**), urine (**B**), and feces (**C**) of piglets fed diets containing control (**CON**) or *Fusarium* toxin contaminated maize (**FUS**) in the absence or presence of mycotoxin inactivating feed additives (**−**/**+**) for 5 weeks. Boxes indicate the 25th and 75th percentile, dashes the medians and whiskers the minimum and maximum values. ANOVA and post hoc Tukey’s test for significant interactions were performed using ranks. Different lower-case letters indicate significant differences between treatment groups (*p* < 0.05). DOM, de-epoxy DON; DONS1, 2, 3, DON sulfonates 1, 2, 3. *n* = 20, 20, and 17 for urine, plasma, and feces of group CON−, respectively; *n* = 18, 19, and 16 for urine, plasma, and feces of group CON +, respectively; *n* = 16, 20, and 12 for urine, plasma, and feces of group FUS−, respectively; *n* = 20, 20, and 17 for urine, plasma, and feces of group FUS +, respectively

DON was the predominant toxin in blood plasma and urine in all feeding groups with clearly higher levels in the FUS-fed groups (*p* < 0.001, Fig. 6). Supplements significantly reduced the DON concentrations in these matrices to a similar extent both in CON+ and FUS+ groups when compared to their unsupplemented counterparts (blood plasma: p_SUP_ = 0.011, urine: p_SUP_ = 0.025) albeit at clearly different DON concentration levels and indicated by non-significant interactions (blood plasma: p_FUS*SUP_ = 0.414, urine: p_FUS*SUP_ = 0.998). DOM remained undetectable in the plasma of CON-fed pigs and was found in only 2 out of 20 samples of group FUS−. In contrast, 8 out of 20 samples were detected positive for DOM in group FUS + at a higher median level explaining the significant interactions between *Fusarium* toxin contamination and supplements. Similar significant relationships were observed for DOM in urine albeit at a generally higher level. In contrast to plasma and urine, DOM levels were clearly higher than DON concentrations in feces. Interestingly, DON concentrations were significantly higher in group FUS + compared to all other groups causing the significant interactions between the fixed factors *Fusarium* toxin contamination and supplements.

The SBS-related DON sulfonates were generally detected at higher concentrations in group FUS + in all 3 matrices and explained the significant interactions between *Fusarium* toxin contamination and supplements except for DONS2 in feces. In group FUS +, DONS2 was the only metabolite detected in plasma, while all 3 DON sulfonates were found in urine. DONS2 was the only detectable DON sulfonate in group FUS− while no sulfonates were found in CON groups in plasma and urine. Besides DONS2 as the predominating DON sulfonate in feces, DONS1 and DONS3 were found in feces of group FUS+, and to a lesser extent in groups FUS− and CON +. DONS2 was also detected in feces of group FUS−, and sporadically in CON groups.

## Discussion

Co-contamination of feed for pigs with DON and ZEN is a common situation in feeding practice and requires control measures to prevent adverse effects in pigs, which are particularly sensitive to both toxins. Large proportions of complete pig feed are manufactured and traded in a dry form which is advantageous from a logistics and storage stability viewpoint. Thus, mycotoxin inactivation can either be managed in combination with feed processing or through supplementing the feed with additives. Usually, a technical treatment for inactivating mycotoxins requires additional technical equipment and specialized manufacturers. In contrast, most commercial feed manufacturers are equipped for the use of feed additives. Based on these advantages and the challenge to inactivate both DON and ZEN in parallel, we used SBS and ZenA as feed additives to test the hypotheses that a combination of both additives would counteract the anorectic DON, and the hyperestrogenic ZEN effects, and that these biological effects are reflected by a lowered systemic availability of both toxins.

Inspecting treatment effects on feed intake first, the 12% decrease of group FUS− compared to group CON− was comparable to the expectations according to a meta-analysis suggesting a decrease in feed intake of approximately 5% for each increase of dietary DON concentration by 1 mg/kg (Döll and Dänicke 2011). Moreover, the decrease in live weight gain was in the same order of magnitude resulting in an unchanged feed-to-gain ratio. This phenomenon has also been reported and was interpreted as an unchanged utilization for live weight gain feeding DON contaminated feed (Goyarts et al. 2005). Based on the adverse effects observed in group FUS−, the changes in group FUS+ are assignable to the additives and showed that negative effects were indeed counteracted. However, as the positive effect of the additives was even more pronounced in group CON+, mechanisms other than counteracting the anorectic effect of DON need to be discussed. Interestingly, feed additives not only stimulated feed intake in both diet types, but live weight gain was increased to a larger extent than feed intake resulting in a significantly improved feed-to-gain ratio by 10% and 8% in groups CON+ and FUS+, respectively. Therefore, nutrient utilization was obviously influenced by the supplements. As ZenA is a very specific additive adjusted to its substrate ZEN and supposed to be active only in the environment of the ingesta, a direct effect on feed and possibly feed intake seems to be less probable. This is supported by the unchanged ZEN concentrations in feed in the course of the experiment proving ZenA’s inactivity in dry feed. In contrast, SBS is an approved food additive used as a preservation agent to prevent microbial spoilage of certain food types (Daniel 1986). Besides their preserving properties, sulfiting agents might exert side effects on food quality since they are known to react with many food constituents. These include, amongst others, aldehydes, ketones, and sulfhydryl moieties, which give rise to the formation of reversible and irreversible adducts with food components, such as sugars, thiamine, nucleic acids, and cysteine residues in proteins (European Food Safety Authority 2016). These properties of sulfiting agents are also the basis of their effects on animal tissues. Lysis of disulfide bonds of proteins and formation of S-sulfonates were described which might provide a hypothetical basis for their potential to interfere with digestion and absorption of nutrients, but also with metabolism. Based on these putative multiple effects both on feed quality and on nutrient utilization, it seems reasonable to assign the positive effects of the supplements on performance to SBS rather than to ZenA.

Literature reports on the effects of SBS on nutrient digestibility are controversial. A significantly increased total tract digestibility of dry matter, gross energy, and protein has been reported for pigs fed a DON-contaminated diet supplemented with 3 g SBS/kg diet compared to the unsupplemented negative control (Mwaniki et al. 2021). The digestibility levels reached those of the positive control which, however, was not tested with supplemental SBS. Therefore, the positive effect of SBS on nutrient digestibility cannot be conclusively assigned to the DON-inactivating effects of SBS. In contrast, total tract digestibility of nutrients remained unaltered in piglets when a DON-contaminated diet was supplemented with SBS (2.5 g Defusion^®^/kg diet) (van Thanh et al. 2015). Again, the control diet was not tested with the additive. Furthermore, it needs to be considered in discussing results observed by using Defusion^®^ that this additive contains many other constituents besides SBS. Apparent ileal digestibility of nutrients was evaluated by employing a complete two by two 2-factorial design where both the positive control and the DON-containing negative control diet were evaluated in the absence and presence of SBS (3 g Defusion^®^/kg diet). Results showed a significantly decreased dry matter and energy digestibility due to SBS addition independent of dietary DON contamination (Bouchard et al. 2019). Taking the discussed experiments on nutrient digestibility together, the positive and unspecific effects of SBS on feed to gain observed in the present experiment cannot be conclusively ascribed to an improved nutrient digestibility although the increased relative pancreas weights of these groups might indicate an involvement of the digestive system.

Besides nutrient digestibility, metabolic effects of SBS need to be considered in interpreting the presumed positive effects of SBS on feed-to-gain ratio and consequently nutrient utilization. In discussing systemic SBS effects, it is important to keep in mind that under physiological conditions (pH 7.4 and 37 °C) sulfiting agents such as SBS and SoS, and also SO_2_, exist as a mixture of the predominant SO_3_^−^ (sulfite) and HSO_3_^−^ (bisulfite), regardless of the introduction of a particular S species into this system. It has been proposed to use the common term “sulfite” for these readily convertible sulfur species (Gunnison 1981).

Interestingly, particularly the metabolic profile of group CON+ was strikingly different compared to the other groups. Blood concentrations of cholesterol, total and direct bilirubin, and GLDH were higher compared to the other treatment groups. Similarly, albumin and calcium concentrations were consistently enhanced both in group CON+ and FUS+, while ALT activity was lower compared to groups CON− and FUS−. Particularly the SBS and SoS related, and *Fusarium* toxin-independent effects on blood albumin and total protein concentrations were repeatedly reported (Tran et al. 2018; Dänicke et al. 2005, 2008). All these changes hint at an involvement of the liver and might reflect its role in sulfite metabolism. This conclusion is further substantiated by the putatively pronounced first-pass metabolism of sulfites (European Food Safety Authority 2016). Consistent with the evaluated static parameters, the liver function appeared to be stimulated by SBS when the ^13^C-methacetin breath test was used as indicator (Dänicke et al. 2008). This compound, labeled with stable isotopes, serves as a substrate for the microsomal CYP1A2 isoform of the P450 superfamily and is rapidly converted to acetaminophen and ^13^CO_2_ in a single dealkylation step (Armuzzi et al. 2002) hinting at the interference of SBS with hepatic xenobiotic metabolism. Interestingly, SBS-related stimulation of in vivo CYP1A2 activity was more pronounced in the presence of SBS-treated DON-contaminated triticale. The latter contained mainly DON sulfonates besides traces of DON suggesting an interaction between SBS and DON sulfonates resulting in a stimulation of CYP1A2 activity. While DON was demonstrated to increase the hepatic mRNA expression of CYP1A2 in pigs (Liu et al. 2022), no related information is available for DON sulfonates and needs to be addressed further.

Parallel to these metabolic alterations, some hematological features were exclusively different between supplemented and unsupplemented groups independently of *Fusarium* toxin contamination. The feed additive-associated lower total leukocyte concentrations were related to lowered banded neutrophils and lymphocytes suggesting a lessened leukocyte turnover and/or stimulated immune system. Moreover, as platelets were influenced in a similar direction, general effects of SBS on bone marrow cannot be excluded. Furthermore, erythrocytes of groups CON+ and FUS+ appeared to be influenced in such a way that they became hyperchromic and macrocytic relative to the unsupplemented groups as indicated by higher MCH and MCV, respectively. Erythrocyte lipid peroxidation, and activities of glucose-6-phosphate dehydrogenase, superoxide dismutases, and glutathione peroxidase were elevated in rats exposed chronically to 25 mg SBS/kg BW via drinking water (Ozturk et al. 2010) underlining the potential of sulfites to interfere with the oxidative and anti-oxidative system. The mean SBS exposure was approximately fourfold higher for the pigs of the present experiment. Whether the described erythrocyte metabolic changes could be associated to morphological alterations needs to be clarified further. The changes in MCH and MCV could further hint at alterations in oxygen transport capacity or efficiency. Whether the significantly lower weights of heart and lungs observed in both supplemented groups could have had consequences for blood circulation and erythrocyte metabolism and turnover cannot be elaborated further based on the present results.

Taken the discussed clinical-chemical and hematological SBS-related changes collectively, it needs to be stressed, however, that parameters varied within or closely around the corresponding reference values (Moritz 2014). Therefore, none of the observed effects can be regarded either as adverse or as non-adverse. Moreover, although no conclusive cause could be identified for the *Fusarium* toxin-independent improvement of performance by the feed additives, its unspecific effects on metabolism and hematology might be related in an unknown way which requires further clarification.

Besides the discussed parameters, scoring of ears, tails, claws, vulvae, and mammary complexes revealed exclusively hints at the estrogenic effects of ZEN as discussed below, but no indications on the swine inflammation and necrosis syndrome (SINS). Particularly, DON is suspected to contribute to this multi-factorial condition (Reiner et al. 2021). However, under the conditions of the present experiment, the predominantly non-inflammatory and non-necrotic lesions recorded for ears, tails, and claws and their time-related changes were probably associated with re-grouping the piglets at the beginning of the experiment. As there were no mycotoxin effects, these traits were consequently not suited to evaluate the efficacy of the supplements.

ZenA is a feed enzyme designed to hydrolyze the highly estrogenic ZEN into derivatives with a low or no estrogenic potential (HZEN, DHZEN). Evaluation of the efficacy of this strategy should consider both the biological effects of ZEN and its reduction in the systemic circulation. Ideally, the uterotropic effect of ZEN as one of the main estrogenic features should be prevented by ZenA which is paralleled by a decreased systemic ZEN bioavailability. In the present experiment, the ZEN concentration of feed for group FUS− was adjusted to a level which was approximately 2.4-fold higher than the critical ZEN concentration of 0.1 mg/kg feed for young female pigs to induce hyperestrogenic effects as a precondition for the evaluation of the efficacy of ZenA. The significant increase in ovary weights of group FUS− was paralleled by a similar trend in uterus weights. Moreover, visual scoring of the mammary complexes of this group revealed the highest prevalence of swelling and reddening, and/or other abnormalities supporting the estrogenic activity of ZEN present in diet FUS−.

In evaluating these estrogenic effects, it should be noted that the dietary ZEN concentration of 0.24 mg/kg was slightly higher than the ZEN concentration of 0.22 mg/kg from a dose–response experiment with the same pig category (Döll et al. 2003) which served as diet level from which European Food Safety Authority (2011) derived a NOEL of 10 µg ZEN/kg BW and subsequently a TDI of 0.25 µg/kg (European Food Safety Authority 2011, 2017). The mean ZEN exposure of piglets of group FUS− also amounted to approximately 10 µg/kg BW and suggests that the current NOEL does not necessarily apply for all situations or is in the range between NOEL and LOEL. Independent of this risk evaluation, it becomes clear that piglets of group FUS+ which were exposed to ZEN at the same order of magnitude as group FUS− failed to develop estrogenic effects underlining the ZEN inactivation efficiency of ZenA. Comparative evaluation of the pattern of ZEN metabolites between group FUS− and FUS+ revealed that the prevention of estrogenic effects in pigs of group FUS+ was accompanied by ZEN levels in blood, urine, and feces similar to those detected in the control groups exposed to trace amounts of ZEN. The systemic bioavailability of ZEN was reviewed to vary between 78 and 87% suggesting that just 22 to 13% of an oral bolus are excreted via feces (Dänicke and Winkler 2015). This high bioavailability clearly suggests effective systemic ZEN absorption in the proximal digestive tract and questioned the biological significance of feces for evaluation ZenA efficiency. Nevertheless, the ZEN hydrolysis pattern in feces clearly resembled that observed in blood and urine. In contrast to feces, urinary excretion of ZEN hydrolysis metabolites can be interpreted as an indicator for the systemic situation. From an analytical viewpoint, it might be even better suited for interpreting ZEN metabolite patterns because of the generally higher concentrations compared to blood. The ZEN hydrolysis-associated decrease in ZEN concentration was paralleled by the detection of significant concentrations of the hydrolysis products HZEN and DHZEN in all 3 matrices. The fact that no or only reduced estrogenic effects (uterus weight, ovary weight, scoring of mammary complex) were detected in group FUS+ at the same time highlights that neither HZEN nor DHZEN were estrogenically active. These results support earlier findings where female pigs exposed to pure HZEN and DHZEN failed to develop hyperestrogenism (Fruhauf et al. 2019).

In contrast to ZEN for which the specific estrogenic effect and its counteraction by ZenA could clearly be assigned to the systemic ZEN residue levels, such an assignment of biological effects of DON to systemic DON and DON sulfonate levels is not possible for the reasons discussed above.

The present experiment and several other studies suggest that SBS and SoS obviously react with DON even during feed mixing (Bouchard et al. 2019; Frobose et al. 2017; Mwaniki et al. 2021) or shortly thereafter whereby this initial decrease occurs more rapidly and is more pronounced at higher moisture contents (Dänicke et al. 2009, 2010; Paulick et al. 2015a; Schwartz-Zimmermann et al. 2014b). Lower moisture contents of ~ 12 to 14%, representing so-called airdry feed, require more time and/or higher doses of sulfiting agents. The initial decrease in DON concentration by approximately 20% was paralleled by the evolvement of DON sulfonates which appeared to increase slightly in the course of the experiment. However, the specific conditions of the current study regarding moisture content, initial DON concentration, and SBS dose prevented a further decrease in DON concentration. It needs to be stressed that the time course of DON and DON sulfonates in feed might be biased by the metabolic fate of modified forms of DON, such as acetylated and glycosylated DON compounds, which could include the cleavage of the side chains and the reaction of either the resulting free DON or the remaining modified forms with SBS. However, this possibility needs to be studied further.

Remarkably, the magnitude of SBS-related DON reduction in feed was closely mirrored by the DON and DONS concentrations in blood and urine. In contrast, DON sulfonates and DOM were the predominating DON metabolites in feces. These differences in DON metabolite profiles between blood/urine and feces suggest an absorption of non-sulfonated DON in the proximal digestive tract. This assumption is supported by earlier findings where DON recovery kinetics in the small intestine of pigs was paralleled by a similar kinetics in the systemic circulation (Dänicke et al. 2004). Moreover, oral bioavailability of DON was reviewed to reach up to 100% suggesting a nearly complete systemic absorption (Dänicke and Brezina 2013). The non-absorbed portion of DON reaching the hindgut, which is quantitatively negligible (Dänicke et al. 2004), along with non-absorbed sulfites gave rise to intensive DON sulfonation putatively due to a longer ingesta retention time and/or an altered intestinal milieu (moisture, pH) including a microbiota involvement. Interestingly, trace concentrations of DONS2 were also detected in feces of group CON− supporting the view that DON sulfonates are common gut-derived DON metabolites in animals (Schwartz-Zimmermann et al. 2014a, 2015; Wan et al. 2014).

The question remains why DON sulfonation obviously occurred only in feed without continuation under the conditions of the upper digestive tract. As sulfites exists predominantly as SO_2_ under acidic conditions (Rose 1993) as physiologically present in the stomach, it has been suggested that these sulfite form would not be available for DON sulfonation (Yu et al. 2021). Thus, these authors tested the stability of SBS encapsulated into hydrogenated palm oil-based microparticles and found a negligible release of SBS from this galenic form when exposed to in vitro stomach conditions while approximately 2/3 of SBS were released into a simulated intestinal fluid. However, the efficacy of this SBS encapsulation needs to be tested in vivo.

When discussing the efficiency of ZEN and DON inactivation comparatively, the differences in kinetics of both toxins need additionally to be considered. Particularly, the more pronounced first-pass effect and enterohepatic-recirculation of ZEN and its metabolites compared to DON (Dänicke and Brezina 2013; Dänicke and Winkler 2015) might increase the intestinal contact time of non-hydrolyzed ZEN with continuously ingested ZenA. However, this remains a hypothesis to be confirmed.

## Conclusions

Although SBS and ZenA were added together, SBS was hypothesized to improve performance and to modify metabolism and hematology independent of dietary *Fusarium* toxin contamination. However, the mechanisms behind these changes could not be clarified and require further consideration.

SBS reduced the DON concentration already in feed by approximately 20% and to the same percentage in blood plasma and urine when groups FUS− and FUS+ were compared, suggesting that no further DON sulfonate formation occurred in the digestive tract before absorbing DON in the upper digestive tract. In contrast, extensive DON sulfonation with the unabsorbed DON portion took place in the hindgut. Though all these changes were significant, a reduction rate of 20% is regarded as insufficient from a practical viewpoint. Based on the literature, the galenic form of SBS application into dry feed needs to be improved to support the DON sulfonation in the proximal digestive tract.

ZenA was shown to be inactive in feed but active in the digestive tract as indicated by the presence of its hydrolyzed low-estrogenic derivatives HZEN and DHZEN both in feces, in systemic circulation, and in the urine of group FUS+ compared to group FUS−. The occurrence of these hydrolysis products was accompanied by a significant decrease in high-estrogenic ZEN concentrations which, in turn, was associated with a decrease in relative weights of uteri and ovaries when compared to group FUS−. Thus, ZenA was proven to be effective, both in terms of biomarkers and biological effects.

## Data Availability

The datasets generated during the current study are available from the corresponding author on reasonable request.
